# Health-Related Quality of Life Among Adults With Multiple Chronic Conditions in the United States, Behavioral Risk Factor Surveillance System, 2007

**Published:** 2010-12-15

**Authors:** Han-Yang Chen, Dennis J. Baumgardner, Jessica P. Rice

**Affiliations:** Center for Urban Population Health, University of Wisconsin School of Medicine and Public Health; Center for Urban Population Health, University of Wisconsin School of Medicine and Public Health, Milwaukee, Wisconsin. Dr Baumgardner is also affiliated with the Aurora UW Medical Group, Milwaukee, Wisconsin; Center for Urban Population Health, University of Wisconsin School of Medicine and Public Health, Milwaukee, Wisconsin

## Abstract

**Introduction:**

Little is known about health-related quality of life (HRQOL) among people with multiple chronic conditions. We examined the association between the number of chronic conditions and self-reported HRQOL outcomes among adults in the United States.

**Methods:**

We used data from the Behavioral Risk Factor Surveillance System (BRFSS) in 2007 (n = 430,912) to compare 4 HRQOL measures for people with any of 8 chronic conditions. We also assessed the frequency of self-reported physical and mental distress and the number of days activity was limited because of chronic conditions. We estimated prevalence and adjusted odds ratios (AORs) and 95% confidence intervals (CIs) by using survey logistic regression analyses.

**Results:**

People with 3 or more chronic conditions had the highest risk of reporting fair or poor health compared with respondents with no chronic conditions (AOR, 8.7; 95% CI, 8.0-9.4). People with cardiovascular conditions or diabetes had higher risk of reporting poor HRQOL outcomes than those with other chronic conditions. The odds ratios for frequent physical distress were consistently higher than those for frequent mental distress and frequent activity limitations for all conditions.

**Conclusion:**

Strategies that help clinicians to manage their patients' chronic conditions may contribute to improved HRQOL among adults. Our findings may help to inform these strategies.

## Introduction

As disease prevention and management improve and the population ages, the prevalence of chronic conditions is accelerating in the United States. Nearly half of adults have at least 1 chronic condition (1), which can result in extended pain and suffering and impaired quality of life.

The growing number of Americans living with chronic illness has shifted the focus of research from treatment and quantity of life to improvement of the quality of life. One of the major goals of *Healthy People 2010* (2) was improving the quality and number of years of healthy life. During the past decade, the research community has increasingly focused on measuring the patient's perspective when evaluating the effect of chronic illness and the benefit of treatment. Self-assessments of health-related quality of life (HRQOL) are rapidly gaining acceptance and are widely used for tracking health status. The Centers for Disease Control and Prevention (CDC) developed a surveillance definition of HRQOL as "perceived physical and mental health over time" (3). Others characterize HRQOL as a subjective assessment of well-being and physical, mental, and social functioning. Thus, HRQOL is recognized as a health-oriented subset of the broader concept of overall quality of life, including aspects of life satisfaction and happiness (4).

The high prevalence of chronic disease in the United States does not tell the whole story. A more specific concern is that many people, especially those in the Medicare population, have multiple chronic conditions (5). Whether HRQOL varies by number of conditions has not been established, despite the research finding that multiple chronic diseases have a substantial negative effect on quality of life, not only how people feel about their lives but also the extent of their psychological distress (6). Some chronic conditions have a stronger relationship with functional impairment than others, but people with more chronic conditions experience more functional impairment and experience it sooner than people with fewer chronic conditions (7).

Our primary objective was to examine the association between the number of chronic conditions and HRQOL outcomes. Our secondary objective was to describe the prevalence of common chronic conditions among the US adult population.

## Methods

We analyzed data from the 2007 Behavioral Risk Factor Surveillance System (BRFSS). BRFSS collects data from ongoing random-digit–dial telephone surveys administered to noninstitutionalized US adults aged 18 years or older on health risk behaviors, preventive health practices, and access to and use of health care services primarily related to chronic conditions. BRFSS data are directly weighted for the probability of selection of a telephone number, the number of adults in a household, and the number of telephones in a household. A final poststratification adjustment is made for nonresponse and noncoverage of households without telephones. The weights for each relevant factor are multiplied to get a final weight (8). In 2007, BRFSS was administered to 430,912 (weighted N = 230,172,178) respondents. The median response rate was 51%, and the median cooperation rate was 72%. A detailed description of the survey design and random sampling procedures is available elsewhere (8). The health sciences institutional review board at the University of Wisconsin-Madison approved this study.

In our analysis, the outcomes of interest were 4 measures of HRQOL from the CDC Healthy Days Core Module (CDC HRQOL-4): general health, mental distress, physical distress, and activity limitations. The CDC HRQOL measures have acceptable content, construct and criterion validity, and test-retest reliability (3,9-12).

In the CDC HRQOL-4, the first question asks respondents to rate their general health on a scale from excellent to poor. We dichotomized these responses as  either "fair/poor" or "good/very good/excellent." The other 3 questions ask about respondents' assessment of their health in the previous 30 days: "How many days was your physical health, which includes physical illness or injury, not good?" (physical distress), "How many days was your mental health, which includes stress, depression, and problems with emotions, not good?" (mental distress), and "How many days did poor physical or mental health keep you from doing your usual activities, such as self-care, work, or recreation?" (activity limitations). We dichotomized these 3 HRQOL variables in terms of their frequency in the previous 30 days (≥14 being frequent or <14 being infrequent). We used the 14-day minimum period because clinicians and clinical researchers often use this period as a marker for clinical depression and anxiety disorders, and longer duration of symptoms is associated with a higher level of activity limitation (13). In addition, most studies based on the BRFSS HRQOL indicators used the same dichotomized criteria as we did (13-16). Thus, our results can be compared with those of previous studies. Moreover, our outcomes of interest were not normally distributed; by dichotomizing the outcomes, we were able to conduct logistic regression analyses without violating the linearity assumption.

We examined respondents by 8 chronic conditions: asthma, arthritis, 3 cardiovascular diseases (heart attack, angina, stroke), diabetes, and hypertension, based on diagnosis of the condition by a health professional, and obesity, defined as a body mass index of at least 30 kg/m^2^, based on self-reported height and weight.

We estimated the prevalence of each chronic condition among adults in the United States. We estimated adjusted odds ratios (AORs) and 95% confidence intervals (CIs) by comparing having each chronic condition with having no condition. To account for potential confounding effects, we controlled for respondents' age, sex, race/ethnicity, education level, income level, employment status, marital status, and health insurance coverage status. In addition, we adjusted for 3 health behavior risk factors: current smoking (defined as ever having smoked at least 100 cigarettes and now smoking every day or some days), current heavy drinking (defined as having more than 2 alcoholic drinks per day for men and having more than 1 alcoholic drink per day for women during the previous 30 days), and no physical activity (defined as not participating in any physical activity during the previous 30 days). To account for complex survey design and produce unbiased estimates of standard errors, we used multivariate survey logistic regression models to estimate AORs and 95% CIs. We conducted all analyses using SAS version 9.2 (SAS Institute, Inc, Cary, North Carolina).

## Results

Approximately 19% of respondents were smokers, 5% were heavy drinkers, and 9% did not participate in any physical activity  (Table 1). Among all survey respondents, 57% reported at least 1 chronic condition. The most prevalent chronic conditions were arthritis (27%), obesity (26%), and hypertension (28%). Among people with cardiovascular diseases, more than 90% of them had 2 or more chronic conditions (Table 2).

The most common conditions for which fair or poor health were reported were cardiovascular diseases (53% for each one) or diabetes (48%) (Table 3). People with 1 or no chronic condition had a higher prevalence of frequent mental distress than frequent physical distress. In contrast, people with 2 or more conditions had a higher prevalence of frequent physical distress than mental distress. Respondents with cardiovascular diseases or diabetes were approximately 7 to 8 times as likely to report fair or poor health as respondents with no chronic condition (Table 4). People with 3 or more chronic conditions were more likely to report poor HRQOL outcomes than those with 1 or 2 conditions. In the population of adults with at least 1 chronic illness, the odds ratios of frequent physical distress varied more widely than those for frequent mental distress and frequent activity limitations across conditions.

## Discussion

Our findings that respondents with multiple chronic conditions reported worse HRQOL than those with 1 or no chronic condition and that frequent physical distress was more common than frequent mental distress were consistent with previous studies in disease-specific populations, such as those of adults with asthma (15), obesity (16), stroke (17), diabetes (18), and arthritis (19).

We found that people without any chronic condition reported a higher prevalence of frequent mental distress than frequent physical distress. However, as the number of chronic conditions increased, frequent physical distress outpaced frequent mental distress. Although our results were consistent with previous findings that the burden of chronic illness is primarily carried in terms of physical health (20), the observation that mental distress is less frequent than physical distress does not imply that mental distress is an unimportant consideration in managing chronic conditions. People with chronic illness may have lived with their conditions for years and feel that they are able to manage their illness and therefore report less mental distress. For example, diabetes patients often rate their well-being positively despite the presence of diabetes-related complications or poor glycemic control (21). These findings suggest that in addition to medical care, the mental health quality of life of the chronically ill population may benefit from social support and be mitigated by socioeconomic status, personality characteristics, and styles of coping with illness.

We found that cardiovascular diseases and diabetes are frequently associated with other surveyed diseases; they may also be associated with many other unmeasured comorbidities. Because our statistical analysis did not adjust for the number of comorbidities, our finding that physical distress is higher in participants with cardiovascular diseases and diabetes may be due to unmeasured comorbidities. In addition, cardiovascular diseases are the primary causes of illness and death among people with diabetes (22) and have a negative effect on quality of life (23).

Our finding of more frequent activity limitations among respondents with at least 1 chronic condition may be a consequence of impaired physical health among the chronically ill population. Physical pain, fatigue, or other limitations may prohibit chronically ill people from engaging in exercise or physical activities. Engaging in such health promotion behaviors, however, and being able to make choices that reflect personal needs and goals are positive characteristics related to quality of life among older adults (24). Thus, applying motivational interviewing techniques (25) to help patients identify their problems and adopt a health-promoting lifestyle early in a disease course, combined with customized medication or treatment that empowers patients to manage their conditions, may improve their quality of life.

Various disease-specific quality-of-life scales have been developed and validated (26-29). Although disease-specific measures provide additional valuable information, they could be more time-consuming than a simple general health questionnaire for respondents to complete. In a general health survey such as BRFSS, a short, valid, generic scale that is applicable across conditions and groups is practical and preferable (30). The CDC HRQOL-4 measures used in BRFSS reflect general HRQOL and compare well against other HRQOL measures, such as the Medical Outcomes Study 36-Item Short-Form Health Survey and the Quality of Well-Being Scale (12,31-33).

Managing chronic illness, especially for people with multiple conditions, presents substantial challenges to professionals in all arenas of health care. Health professionals seek not only to develop better strategies to manage chronic disorders and prevent complications but also to maintain or enhance the functional abilities of people who are chronically ill. Clinician awareness of patients' needs early in their care may reduce the effect of chronic comorbidities on HRQOL. By targeting outcomes that patients seem to value most, clinicians could provide customized treatment plans that patients are more motivated to follow. Thus, a better understanding of HRQOL related to chronic conditions may lead to more effective preventive education and improved care of patients with chronic illness.

Our study had several limitations. First, BRFSS does not survey people who are hospitalized or institutionalized. People with severe conditions might not have been able to answer the telephone or be interviewed. For example, stroke survivors interviewed through BRFSS may have less severe disabilities than the total population of stroke survivors. BRFSS also excludes people with no telephones or people who use only cellular telephones. People who use only cellular telephones tend to be younger and may have fewer chronic conditions (34), whereas people with no telephones are usually from a lower socioeconomic group, which is associated with poor HRQOL (35). Thus, BRFSS may either underestimate or overestimate the prevalence of people with impaired physical or mental health. Second, the analyses were based on self-reported data, which may be influenced by reporting bias. However, results from previous validation studies showed substantial agreement between self-reported disease status and disease status as documented in medical records (36). Third, since BRFSS did not include questions about the severity of impairment resulting from conditions or comorbidities, we were unable to assess the association between severity of impairment and HRQOL. It is possible that people who report better physical health or fewer activity limitations had a less severe impairment from their conditions than those who reported worse HRQOL. However, in our analyses, we were able to categorize respondents by the number of conditions they had and to assess the association with self-reported HRQOL. Finally, the cross-sectional study design allowed us to demonstrate only an association. Future studies using a longitudinal design are necessary to assess the temporal sequence of the onset of the chronic conditions and the change in HRQOL.

Despite these potential limitations, our findings suggest that HRQOL varies substantially by the category and number of chronic conditions. The prevalence and AORs of frequent physical distress vary more widely across the chronic conditions and appear to be higher than those of frequent mental distress; HRQOL consistently decreases as the number of conditions increases. Strategies by individual clinicians and teams providing customized medication or treatment to improve the HRQOL of their patients should focus on preventing sequelae and comorbidities of the patient's chronic disease and targeting the areas that the patient values most, such as the ability to perform daily activities, a desired recreational activity, or playing with grandchildren. Motivating patients to take charge of their disease management and adopt healthy lifestyles that improve physical health may improve their HRQOL. On a broad scale, health care organizations could focus care management resources on enhancing communication with patients and guiding them in making choices to improve their health and HRQOL.

## Figures and Tables

**Table 1:** Sample Characteristics, Behavioral Risk Factor Surveillance System (n = 430,912), United States, 2007

**Characteristic**	Weighted %^a^
**Age, y**	
18-44	50
45-64	33
≥65	17
**Sex**	
Men	49
Women	51
**Race/ethnicity**	
Non-Hispanic white	69
Non-Hispanic black	10
Hispanic	15
Other	6
**Education**	
Less than high school	12
High school diploma	29
More than high school	60
**Annual household income, $**	
<25,000	22
25,000-49,999	23
50,000-74,999	15
≥75,000	27
Don't know/not sure/refused	13
**Employment status**	
Employed	61
Unemployed	5
Homemaker/student	13
Retired	16
Unable to work	5
**Health insurance coverage**	
No	15
Yes	85
**Marital status**	
Married	61
Single, previously married	18
Single, never married	18
Member of an unmarried couple	4
**Smoking behavior**	
Current smoking^b^	19
No current smoking	81
**Drinking behavior**	
Heavy drinking^c^	5
No heavy drinking	95
**Physical activity behavior**	
No physical activity^d^	9
Some physical activity	91

a Weighted N = 230,172,178.

b Current smoking defined as ever having smoked at least 100 cigarettes and now smoking every day or some days.

c Heavy drinking defined as more than 2 alcoholic drinks per day for men and more than 1 alcoholic drink per day for women during the previous 30 days.

d No physical activity defined as not participating in any physical activity during the previous 30 days.

**Table 2:** Prevalence of Chronic Conditions, Behavioral Risk Factor Surveillance System (n = 430,912), United States, 2007

Condition^a^	Overall, %	1 Condition, %	2 Conditions, %	≥3 Conditions, %
**Any**	57	50	27	23
**Asthma**	8	33	28	39
**Arthritis**	27	30	31	39
**Cardiovascular disease**				
Myocardial infarction	4	6	16	78
Angina	4	6	15	78
Stroke	3	9	19	73
**Diabetes**	9	11	23	67
**Obesity**	26	37	29	34
**Hypertension**	28	25	34	41

a Respondents were categorized as having a condition if they had ever been diagnosed with it by a health professional or, in the case of obesity, if their body mass index (calculated from self-reported weight and height) was ≥30 kg/m^2^.

**Table 3:** Prevalence of Health-Related Quality of Life Outcomes, by Chronic Conditions, Behavioral Risk Factor Surveillance System (n = 430,912), United States, 2007

**Condition^a^**	Fair or Poor Health, %	Frequent Physical Distress,^b^ %	Frequent Mental Distress,^b^ %	Frequent Activity Limitations,^b^ %
**Asthma**	30	23	19	15
**Arthritis**	31	23	15	14
**Cardiovascular disease**				
Myocardial infarction	53	34	17	21
Angina	53	35	18	22
Stroke	53	38	20	24
**Diabetes**	48	28	16	17
**Obesity**	25	16	13	10
**Hypertension**	31	20	13	12
**Number of conditions**				
0	7	4	7	3
1	14	9	10	6
2	24	16	13	10
≥3	47	32	18	20

a Respondents were categorized as having a condition if they had ever been diagnosed with it by a health professional or, in the case of obesity, if their body mass index (calculated from self-reported weight and height) was ≥30 kg/m^2^.

b On ≥14 days of the preceding 30 days.

**Table 4:** Health-Related Quality of Life Outcomes, by Chronic Conditions, Behavioral Risk Factor Surveillance System (n = 430,912), United States, 2007^a^

**Condition^b^**	Fair or Poor Health, AOR (95% CI)	Physical Distress,^c^ AOR (95% CI)	Mental Distress,^c^ AOR (95% CI)	Activity Limitations,^c^ AOR (95% CI)
**No condition**	1 [Reference]	1 [Reference]	1 [Reference]	1 [Reference]
**Asthma**	4.7 (4.3-5.1)	3.9 (3.6-4.3)	2.3 (2.2-2.6)	3.1 (2.8-3.5)
**Arthritis**	4.5 (4.2-4.8)	4.0 (3.7-4.3)	2.5 (2.3-2.6)	3.3 (3.0-3.6)
**Cardiovascular disease**				
Myocardial infarction	8.3 (7.6-9.2)	4.8 (4.4-5.3)	2.5 (2.3-2.8)	3.9 (3.5-4.4)
Angina	9.2 (8.4-10.0)	5.4 (4.9-6.0)	2.8 (2.5-3.1)	4.2 (3.8-4.7)
Stroke	6.9 (6.2-7.7)	4.8 (4.3-5.4)	2.5 (2.2-2.9)	3.7 (3.3-4.2)
**Diabetes**	7.6 (7.0-8.3)	4.2 (3.8-4.5)	2.3 (2.1-2.5)	3.1 (2.8-3.4)
**Obesity**	3.5 (3.3-3.8)	2.7 (2.5-2.9)	1.8 (1.7-2.0)	2.4 (2.2-2.6)
**Hypertension**	4.3 (4.0-4.6)	3.1 (2.8-3.3)	2.0 (1.9-2.2)	2.7 (2.4-2.9)
**Number of conditions**				
1	2.1 (1.9-2.3)	1.9 (1.7–2.0)	1.5 (1.4-1.6)	1.7 (1.6-1.9)
2	3.7 (3.4-4.0)	3.0 (2.8-3.3)	2.1 (1.9-2.2)	2.5 (2.3-2.8)
≥3	8.7 (8.0-9.4)	5.5 (5.1-5.9)	2.9 (2.7-3.1)	4.1 (3.8-4.5)

Abbreviations: AOR, adjusted odds ratio; CI, confidence interval.

a Adjusted by age, sex, race/ethnicity, education, income, employment, health insurance coverage status, marital status, and 3 risk behaviors: smoking, heavy drinking, and no physical activity. All AORs are significant at *P* < .001.

b Respondents were categorized as having a condition if they had ever been diagnosed with it by a health professional or, in the case of obesity, if their body mass index (calculated from self-reported weight and height) was ≥30 kg/m^2^.

c On ≥14 days of the preceding 30 days.
